# The Monitoring of Accumulations of Elements in Apple, Pear, and Quince Fruit Parts

**DOI:** 10.1007/s12011-024-04223-3

**Published:** 2024-05-15

**Authors:** Isam Ali Mohamed Ahmed, Mehmet Musa Özcan, Fahad AlJuhaimi, Zainab Albakry

**Affiliations:** 1 Department of Food Science & Nutrition, College of Food and Agricultural Sciences, King Saud University, Riyadh, Saudi Arabia; 2 Faculty of Agriculture, Department of Food Engineering, Selcuk University, 42031 Konya, Turkey; 3 College of Ocean Food and Biological Engineering, Jimei University, Xiamen, 361021 China

**Keywords:** Fruit parts, Drying, Macro and micro elements, ICP-OES

## Abstract

In this study, the distribution of biogenic macro and micro element contents in the peel, pulp, and seeds of some cultivated fruits was observed. The element concentrations of these fruits, which have high commercial value and consumption in the world, were analyzed with ICP-OES. In the “Golden” and “Starking” apple varieties, the lowest and highest calcium amounts were detected in the pulp and seed parts of the fruits, respectively. Additionally, the lowest and highest calcium amounts of pear and quince fruits were found in the seed and pulp and peel and seed parts of the fruits, respectively. Potassium amounts of “Golden” and “Starking” apple parts were established to be between 3585.82 (seed) and 3930.87 mg/kg (pulp) and 3533.82 (peel) and 5671.55 mg/kg (pulp), respectively. Potassium amounts of pear and quince fruit parts were measured to be between 2340.65 (seed) and 5405.97 mg/kg (pulp) and 4455.23 (seed) and 8551.12 mg/kg (pulp), respectively. Iron quantities of the parts of “Golden” and “Starking” apple fruits were established from 4.80 (pulp) and 17.14 mg/kg (seed) to 7.80 (pulp) and 14.53 mg/kg (peel), respectively. While the Fe quantities of pear fruit parts are found to be between 4.51 (pulp) and 15.40 mg/kg (peel), the Fe contents of the parts of quince fruits were determined to be between 5.59 (pulp) and 27.27 mg/kg (peel). Zinc quantities of the parts of pear and quince fruits were recorded to be between 8.43 (pulp) and 12.71 mg/kg (seed) and 0.96 (pulp) and 37.82 mg/kg (seed), respectively. In fruit parts, the highest element was found in the seed, followed by pulp and peel in decreasing order.

## Introduction

The vast majority of minerals, which are considered important nutrients for human health, are provided by foods and include macro (K, Mg, Na, Ca, and P) and micro elements (Mn, Zn, Fe, and Cu), which play an important role in various biological processes important for humans [1–3]. Mineral deficiencies in humans can cause organ damage and metabolic disorders that can lead to acute and chronic diseases and even fatal cases [4, 5]. Therefore, fruits and vegetables have an important place in human nutrition in terms of minerals, vitamins, and other phytochemicals [6, 7]. Factors affecting trace element concentrations in fruits are agricultural practices such as the mineral composition of the soil where they are grown, the composition of irrigation water, weather conditions, and the type and amount of fertilizers used [8, 9]. Fruits, which constitute a special source of essential nutrients necessary for human health, significantly reduce both chronic and malnutrition-related diseases such as diabetes and obesity [10, 11]. Fruits, which can be considered a good source of nutrients and food supplements, are known to be excellent sources of nutrients such as minerals and vitamins, especially potassium, calcium, and magnesium. In order to live a good and healthy life, the necessary amounts of these elements must be taken in the daily human diet. Calcium, which is known to delay ripening and aging, reduce storage disorders, and is found in the tissues of many fruits, has been reported to be an important factor determining fruit storage quality [12, 13]. Since trace elements are essential components of enzyme systems, when a trace element is deficient, a characteristic syndrome is produced that reflects the specific functions of the nutrient in the organism’s metabolism. Simple or conditional deficiencies of minerals therefore have profound effects on metabolism and tissue structure [14]. Minerals that cannot be synthesized by the human body can be obtained by consuming some foods. Preservation and drying techniques make other nutritional elements, especially minerals, more concentrated [15]. Minerals that are necessary for enzyme activity and therefore play a role in various metabolic activities in the human body are found in large amounts in dried fruits, and some of these minerals are necessary for the proper functioning of the body and enzyme activity in humans [16]. Wild and cultivated fruits, which are the main source of minerals, are excellent sources of essential minerals and have significant effects on human metabolism due to their different nutritional compositions, and various types of wild fruits are highly nutritious and have a wide range of uses in human nutrition [17, 18]. Due to continuous progress in techniques for the quantitative analysis of biogenic elements, natural foods are understood to be important sources of minerals. The aim of this study was to observe the distribution of biogenic macro and micro element contents in the peel, pulp, and seeds of some cultivated fruits.

## Material and Methods

### Material

**Fig. 1:**
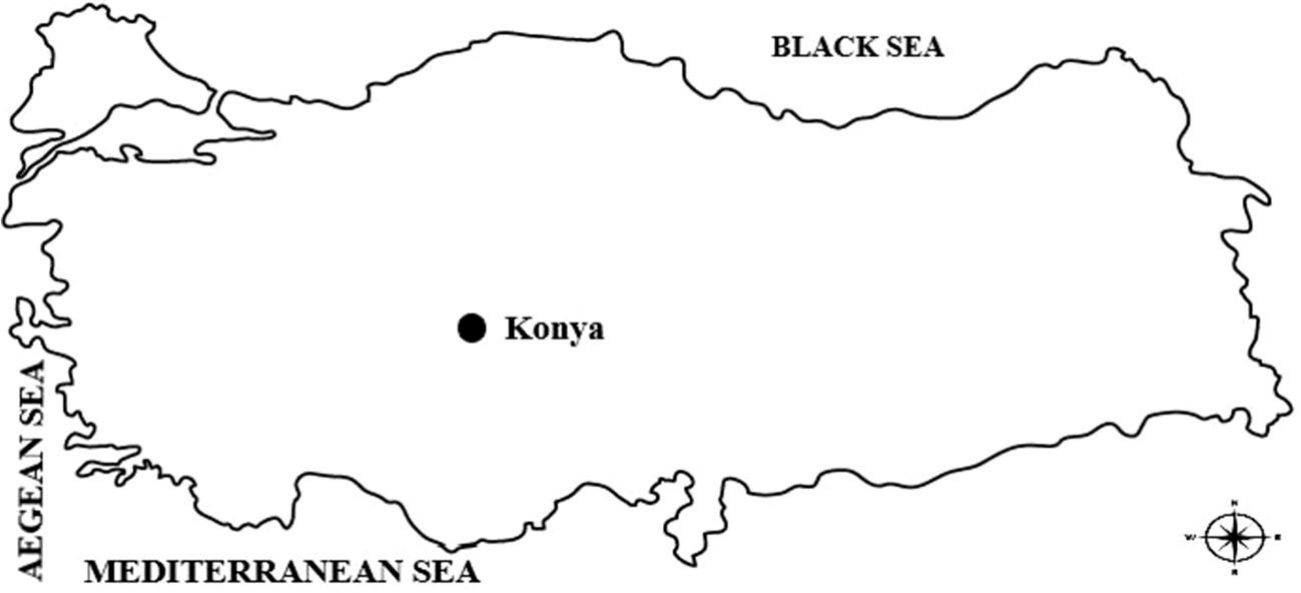
Locations where the fruits (apples, pear, and quince) used in this study were collected

Fruit samples (apples (Golden and Starking cv.), pear, and quince) were provided from Konya province in 2023. Before the analysis, the fruits were washed with distilled water and then dried in a shaded place. HNO_3_ and H_2_O_2_ are analytical grades (Merck Company, Darmstadt, Germany). The location where the fruits (apples, pear, and quince) used in this study is shown in Fig. 1.

### Method

#### Drying Process of Fruits

Fresh fruit samples (apples, pear, and quince) were washed with distilled water and dried, and peels, pulp, and seeds were separated with a knife. After the parts for fruits were divided into three separate drying groups, drying processes were carried out in the shade until they reached a constant weight.

#### Element Contents of Fruit Parts

After 0.2 g of fruit parts were burned in a microwave device at 210 °C and 200 PSI pressure in 5 mL of concentrated HNO_3_ and 2 mL of H_2_O_2_ (30% w/v), the volumes of the dissolved samples were completed to 20 mL with deionized water. Then, element concentrations in the fruit samples were analyzed with ICP-OES [19].

Working conditions of ICP-OES:

o Instrument: ICP-OES (Agilent 5110; Germany)
o RF Power: 0.7–1.5 kw (1.2–1.3 kw for axial)
o Plasma gas flow rate (Ar): 10.5–15 L/min (radial), 15 (axial)
o Auxilary gas flow rate (Ar): 1.5
o Viewing height: 5–12 mm
o Copy and reading time: 1–5 s (max. 60 s)
o Copy time: 3 s (max. 100 s)

Wavelengths, detection LOD (mg/kg), limit of quantification (LOQ) (mg/kg), and coefficient of determination (*R*^2^) of elements analyzed with ICP-OES are shown in Table 1.

**Table 1:** Wavelengths, detection LOD (mg/kg), limit of quantification (LOQ) (mg/kg), and coefficient of determination (*R*^2^) of elements

Elements	Wavelengths (nm)	Detection LOD (mg/kg)	Limit of quantification (LOQ) (mg/kg)	Coefficient of determination (*R*^2^)
B	249.772	0.118227	0.394091	0.999
Ca	317.933	259.2863	864.2877	0.999
Cu	327.395	0.027818	0.092727	0.999
Fe	238.204	1.411773	4.705909	0.999
K	766.491	131.6843	438.9477	0.999
Mg	279.553	58.98845	196.6282	0.999
Mn	257.610	0.424227	1.414091	0.999
P	213.618	4.346591	14.48864	0.999
Zn	213.857	0.208636	0.695455	0.999

### Statistical Analysis

The mineral analysis was performed in triplicate for each sample. Results were expressed as the mean value ± standard deviation (mg/kg DW). The JMP statistical program was used for the statistical analysis of the results obtained. Statistical differences were determined by the analysis of variance (ANOVA) procedure in all data (*p* < 0.01 and *p* < 0.05) [20]. In order to examine the correlation between different fruit varieties (Golden apple, Starking apple, pear, and quince), a multivariate cluster analysis was carried out using the PAST statistical program to perform principal component analysis (PCA) [21].

## Results and Discussion

### Macro and Micro Element Contents of the Peel, Pulp, and Seeds of the Fruits

Macro and micro element contents of the peel, pulp, and seeds of three different fruit species belonging to the Rosaceae family (*Malus communis*, *Pyrus communis* L., and *Cydonia oblonga* Mill.) are assigned in Table 2. The macro and micro elements established in the highest amounts in fruit parts were K, Ca, Mg, Cu, P, Fe, and Zn. Calcium amounts of “Golden” and “Starking” apple varieties were recorded to be between 1191.06 (pulp) and 2765.70 mg/kg (seed) and 1195.69 (pulp) and 3016.74 mg/kg (seed), respectively. While Ca values of pear parts vary between 1252.49 (seed) and 1839.27 mg/kg (peel), calcium quantities of quince parts were recorded to be between 425.67 (pulp) and 2283.60 mg/kg (seed), while K amounts of “Golden” apple parts are established to be between 3585.82 (seed) and 3930.87 mg/kg (pulp), potassium quantities of “Starking” apple parts were recorded to be between 3533.82 (peel) and 5671.55 mg/kg (pulp). Potassium amounts of pear and quince fruit parts were measured to be between 2340.65 (seed) and 5405.97 mg/kg (pulp) and 4455.23 (seed) and 8551.12 mg/kg (pulp), respectively. While P quantities of “Golden” apple fruit parts vary between 873.03 (pulp) and 4129.80 mg/kg (seed), phosphorus quantities of “Starking” apple fruit parts were determined to be between 978.44 (pulp) and 4810.90 mg/kg (seed). Also, phosphorus values of the parts of pear “Deveci cv” and quince fruits were recorded to be between 855.49 (peel) and 2296.25 mg/kg (seed) and 1223.06 (pulp) and 4216.02 mg/kg (seed), respectively.

**Table 2:** Macro element contents in the peel, pulp, and seeds of some cultivated fruits dried in the shade (mg/kg)

Fruits	K				P				Ca				Mg			
	Peel	Pulp	Seed	Mean	Peel	Pulp	Seed	Mean	Peel	Pulp	Seed	Mean	Peel	Pulp	Seed	Mean
Golden apple	3905.30 E	3930.87 E	3585.82 F	3807.33 D	975.75 EF	873.03 F	4129.80 B	1992.86 B	1372.17 EF	1191.06 FG	2765.70 B	1776.31B	464.58 E	312.25 F	2473.27 B	1083.36 B
Starking apple	3533.82 F	5671.55 B	3928.08 E	4377.81 B	854.04 F	978.44 EF	4810.90 A	2214.46 A	1533.56	1195.69 FG	3016.74 A	1915.33 A	586.12 D	317.62 F	2752.07 A	1218.60 A
Pear	4763.32 C	5405.97 B	2340.65 G	4169.98 C	855.49 F	1028.92 E	2296.25 C	1393.55 C	1839.27 D	1354.57 EF	1252.49 F	1482.11 C	483.71 E	463.74 E	1386.40 C	778.95 C
Quince	4645.31 CD	8551.12 A	4455.23 D	588.89 A	1263.65 D	1223.06 D	4216.02 B	2234.24 A	1033.1	425.67 H	2283.60 C	1247.46 D	466.52 E	267.28 F	2804.06 A	1179.29 A
Mean	4211.94 B	5889.87 A	3577.45 C		987.23 B	1025.86 B	3863.24 A		1444.53 B	1041.75 C	2329.63 A		500.23 B	340.22 C	2354.70 A	

A–G: *p *< 0.01

### Micro Element Contents of the Peel, Pulp, and Seeds of the Fruits

Micro element contents of the peel, pulp, and seeds of three different fruit species belonging to the Rosaceae family (*Malus communis*, *Pyrus communis* L*.*, and *Cydonia oblonga* Mill.) are given in Table 3. As micro elements, the iron quantities of the parts of “Golden” and “Starking” apple fruits were established to be between 4.80 (pulp) and 17.14 mg/kg (seed) and 7.80 (pulp) and 14.53 mg/kg (peel), respectively. While iron amounts of pear fruit parts are found to be between 4.51 (pulp) and 15.40 mg/kg (peel), the Fe quantities of the parts of quince fruits were assessed to be between 5.59 (pulp) and 27.27 mg/kg (peel). In addition, zinc amounts of “Golden” and “Starking” apple fruit parts were established between 6.76 (pulp) and 22.08 mg/kg (seed) and 7.35 (pulp) and 22.85 mg/kg (seed), respectively. Also, zinc quantities of the parts of the pear and quince fruits were recorded to be between 8.43 (pulp) and 12.71 mg/kg (seed) and 0.96 (pulp) and 37.82 mg/kg (seed), respectively. Cu content was found in the highest amounts in the core parts of the fruits. The seeds of the “Golden” apple, “Starking” apple, pear, and quince fruits contained 9.07, 11.80, 10.39, and 12.97 mg/kg Cu, respectively. In addition, the lowest B elements and Cu, Mn, P, Mg, and Zn were detected in the pulp parts of the fruits. The highest amount of B element was recorded in the seeds of the fruits. While most of the elements were generally accumulated in the seed parts of the fruits, the amounts of elements were found at low levels in the fruit pulp. Therefore, in fruit parts, the highest element was found in the seed, followed by pulp and peel in decreasing order. Amounts of elements were found in different concentrations in fruit parts. This may be due to the genetic structure, species, variety, transportation conditions, storage factors, environmental factors, elemental content of the soil where the fruits grow, and the physiological status of the plants.

**Table 3:** Macro and micro element contents in the peel, pulp, and seeds of some cultivated fruits dried in the shade (mg/kg)

Fruits	Mn				Cu				Fe				B				Zn			
	Peel	Pulp	Seed	Mean	Peel	Pulp	Seed	Mean	Peel	Pulp	Seed	Mean	Peel	Pulp	Seed	Mean	Peel	Pulp	Seed	Mean
Golden apple	3.89 F	0.96 I	23.23 B	9.36 B	2.91 G	1.91 H	9.07 D	4.63 C	16.51 CD	4.80 G	17.14 C	12.82 B	9.72 D	6.74 F	16.15 C	10.87 B	6.79 F	6.76 F	22.08 B	11.80 B
Starking apple	7.71 E	2.13 H	27.85 A	12.56 A	2.90 G	2.73 G	11.80 B	5.81 B	14.53 D	7.80 F	11.74 E	11.29 C	9.65 D	6.66 F	16.38 C	10.90 B	7.73 EF	7.35 F	22.85 B	12.64 A
Pear	4.40 F	1.81 H	10.47 D	5.56 D	6.41 E	5.48 F	10.39 C	7.43 A	15.40 CD	4.51 G	4.79 G	8.23 D	7.60 E	5.71 G	3.20 H	5.51 C	11.64 D	8.43 E	12.71 C	10.93 C
Quince	2.84 G	0.98 I	21.24 C	8.39 C	2.80 G	1.91 H	12.97 A	5.89 B	27.27 A	5.59 FG	25.01 B	19.29 A	18.28 B	9.83 D	19.86 A	15.99 A	1.25 G	0.96 G	37.82 A	13.20 A
Mean	4.71 B	1.47 C	20.72 A		3.75 B	3.01 C	11.06 A		18.43 A	5.62 C	14.67 B		11.31 B	7.24 C	13.90 A		6.85 B	5.88 C	23.76 A	

A–G: *p* < 0.01

Özcan et al. [22] reported that hawthorn fruits contained 3046.37 Ca, 13,531.96 K, 1502.55 Mg, 312.18 Na, and 1477.88 mg/kg P. In other studies, the calcium quantities of peaches were recorded to be between 5.1 and 9.12 mg/100 g [23]. The apple samples contained 1.75 to 8.74 mg/100 g Ca and 0.003–0.007 mg/100 g [24]. Plum (Chile) and strawberry (Belgium) fruits contained 3.7 and 6.25 mg/100 g Na, 154 and 115 K, 1.05 and 1.45 Ca, 3.1 and 3.6 Mg, 0.28 and 0.255 Cu, 0.095 and 0.1825 Fe, and 0.08 and 0.05 mg/100 g Zn, respectively [25]. Sour cherry (Bucovat) and cherry (Bulgaria) fruits contained 3.8 and 3.95 Na, 156 and 134.5 K, 2.5 and 0.9 Ca, 5.7 and 3.15 Mg, 0.13 and 0.065 Mn, 0.17 and 0.09 Cu, and 0.13 and 0.065 Fe mg/100 g [25]. Cristina et al. [25] determined 4.95 mg/100 g Na, 165.5 K, 0.85 Ca, 2.95 Mg, 0.275 Cu, 0.075 Mn, 0.0375 Fe, and 0.02 mg/100 g Zn in rape fruit (Egypt). Sun drying, sulfured, and sweet apricot samples contained 207–218 mg/100 g Ca, 132–141 mg/100 g Mg, 20.47–21.82 mg/100 g Na, 128.75–156.63 mg/100 g Mn, 100.71–180.23 ppm, 54.56–64.50 ppm Cu, and 52.66–63.21 ppm Zn Fe [26]. Aladag et al. [7] determined P, K, Ca, Mg, and S in hawthorn and wild pear fruits. Mohammed et al. [27] determined 8.48–13.22 g/100 g Ca, 6.01–7.99 g/100 g P, 0.015–0.083 g/100 g Fe, 0.008–0.032 g/100 g Mn, 0.110–0.162 g/100 g Zn, and 0.022–0.039 g/100 g Cu in fresh and dried mango fruits. In addition, it was observed that drying air speeds increased the concentrations of some elements (P and K) and decreased the content of some elements such as Ca, Mg, Fe, and Zn [28]. Although 20–25 mg of iron is taken with a normal daily diet, only 1–2 mg of iron can be absorbed from the small intestines [29]. People’s daily zinc needs vary depending on their age, gender, and health status. After the iron mineral, which ensures the functioning of tissues and organs in the body, the most important trace element is known as zinc. The total amount of zinc, which plays an important role in the immune system and metabolic activities, is estimated to be 2 g in adult individuals. More than 80% of it is found in bone, muscle, hair, and skin. For a strong nervous system and immune system, sufficient zinc intake must be provided to the body [30]. Zinc, which has important physiological effects on plants and animals and plays a role in many biological functions, is one of the essential trace elements and a micronutrient that is important in human nutrition. Zinc is the second-most abundant trace element in the human body after iron and is necessary for the function of more than 300 enzymes in the body [31]. It is therefore important to optimize the drying process to ensure the good chemical composition of a dried product. The drying technique depends on various factors such as the required product type, size, maturity level, structure, color, aroma, chemical composition, and nutritional composition, as well as the expected final quality, dryer availability, and costs [28]. In recent studies, when the biogenic element contents of fruits were compared depending on their parts, some important differences were detected depending on the fruit parts. These differences may vary depending on the nutrient content of the soil where the fruits are grown, irrigation water, harvest time, location, drying method, temperature, light intensity, and fruit types, as well as the different parts of the fruits. When the biogenic element contents of fruit parts were compared with literature data, they were mostly compared with fruit pulps, and partial differences were observed. No detailed study including fruit seeds and peels has been found. Interestingly, the majority of elemental accumulation in fruits is determined in the seed.

### Principal Component Analysis (PCA)

**Fig. 2:**
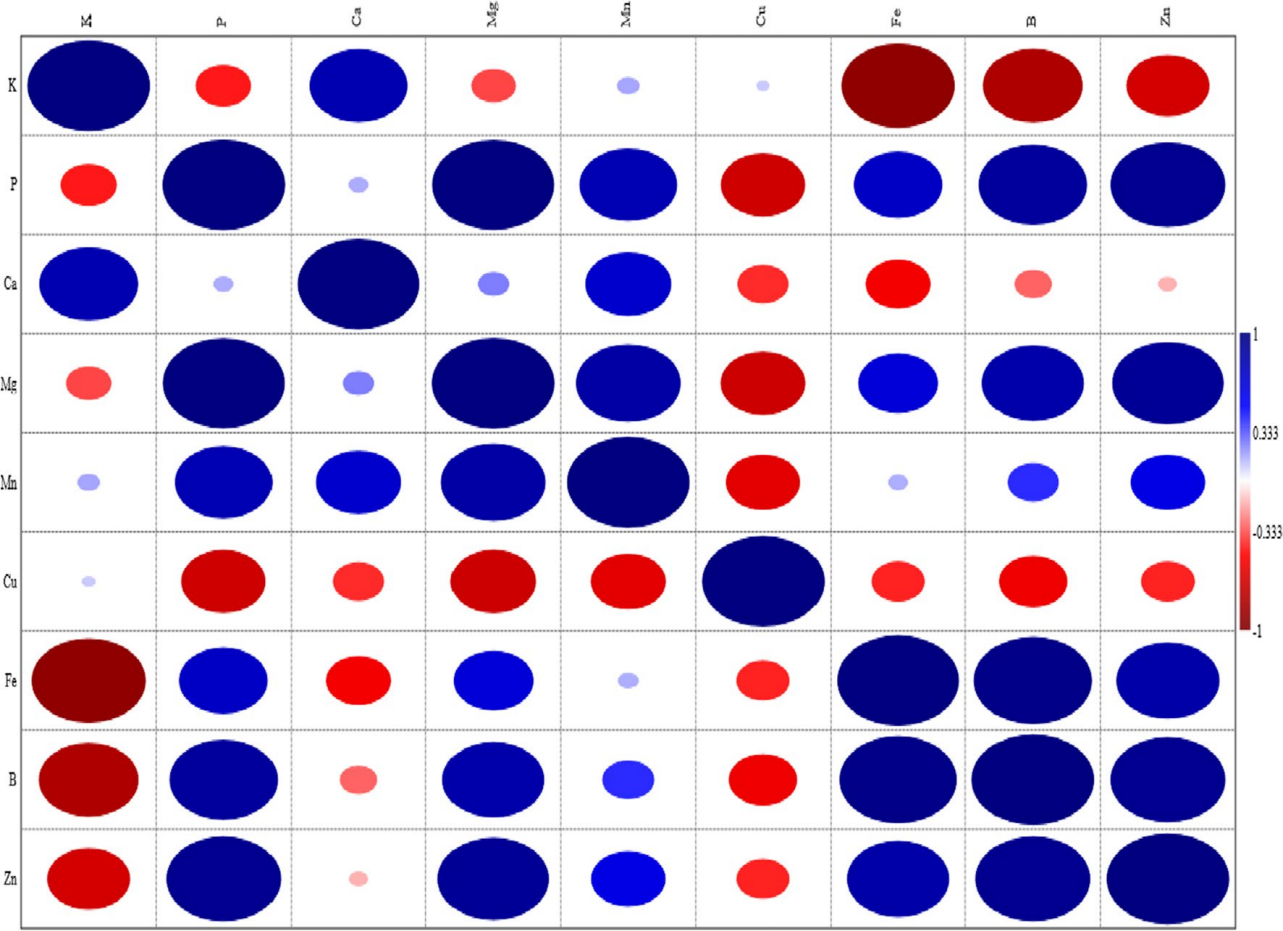
Pearson’s correlation (*r*) between macro (K, P, Ca, and Mg) and micro (Mn, Cu, Fe, B, and Zn) nutritional element contents of different fruit varieties (Golden apple, Starking apple, pear, and quince)

The Pearson correlation (*r*) between macro (K, P, Ca, and Mg) and micro (Mn, Cu, Fe, B, and Zn) nutritional element contents of different fruit varieties (Golden apple, Starking apple, pear, and quince) is shown in Fig. 2. As can be seen from examining Fig. 2, although there are positive and negative relationships between the nutritional elements of fruit varieties, it was determined that the relationship between P quantities and Mg contents was a significant and highly positive one (*p* < 0.05, *r* > 0.70). Similarly, this study found that the Fe contents of fruit varieties showed significant and highly positive relationships with their B contents. The Pearson correlation analysis conducted in this study aimed to determine the strength and direction of the relationship between different fruit varieties and their nutritional element contents. Thus, the Pearson correlation tried to draw the best-fit line on the data of different fruit varieties and nutritional element contents, and the Pearson correlation coefficient (*r*) revealed how far all these data points were from the best-fit line [32].

## Conclusion

The lowest B elements and Cu, Mn, P, Mg, and Zn were detected in the pulp parts of the fruits. The highest amount of B element was recorded in the seeds of the fruits. While most of the elements were generally accumulated in the seed parts of the fruits, the amounts of elements were found at low levels in the fruit pulp. Therefore, in fruit parts, the highest element was found in the seed, followed by pulp and peel in decreasing order.

## Data Availability

No datasets were generated or analyzed during the current study.
